# Isoglutaminyl cyclase contributes to CCL2-driven neuroinflammation in Alzheimer’s disease

**DOI:** 10.1007/s00401-015-1395-2

**Published:** 2015-02-11

**Authors:** Maike Hartlage-Rübsamen, Alexander Waniek, Juliane Meißner, Markus Morawski, Stephan Schilling, Carsten Jäger, Martin Kleinschmidt, Holger Cynis, Astrid Kehlen, Thomas Arendt, Hans-Ulrich Demuth, Steffen Roßner

**Affiliations:** 1 Paul Flechsig Institute for Brain Research, University of Leipzig, Jahnallee 59, 04109 Leipzig, Germany; 2 Department of Drug Design and Target Validation MWT Halle/Saale, Fraunhofer Institute for Cell Therapy and Immunology IZI Leipzig, Biocenter, Weinbergweg 22, 06120 Halle (Saale), Germany; 3 Institute for Medical Microbiology, University Hospital of the Martin Luther University, Halle/Saale, Germany

## Abstract

The brains of Alzheimer’s disease (AD) patients are characterized by deposits of Abeta peptides and by accompanying chronic inflammation. Here, we provide evidence that the enzyme isoglutaminyl cyclase (isoQC) is a novel factor contributing to both aspects of AD pathology. Two putative substrates of isoQC, N-truncated Abeta peptides and the monocyte chemoattractant chemokine CCL2, undergo isoQC-catalyzed pyroglutamate (pGlu) modification. This triggers Abeta aggregation and facilitates the biological activity of CCL2, which collectively results in the formation of high molecular weight Abeta aggregates, glial cell activation, neuroinflammation and neuronal cell death. In mouse brain, we found isoQC to be neuron-specifically expressed in neocortical, hippocampal and subcortical structures, localized to the endoplasmic reticulum and Golgi apparatus as well as co-expressed with its substrate CCL2. In aged APP transgenic Tg2576 mice, both isoQC and CCL2 mRNA levels are up-regulated and isoQC and CCL2 proteins were found to be co-induced in Abeta plaque-associated reactive astrocytes. Also, in mouse primary astrocyte culture, a simultaneous up-regulation of isoQC and CCL2 expression was revealed upon Abeta and pGlu-Abeta stimulation. In brains of AD patients, the expression of isoQC and CCL2 mRNA and protein is up-regulated compared to controls and correlates with pGlu-Abeta load and with the decline in mini-mental state examination. Our observations provide evidence for a dual involvement of isoQC in AD pathogenesis by catalysis of pGlu-Abeta and pGlu-CCL2 formation which mutually stimulate inflammatory events and affect cognition. We conclude that isoQC inhibition may target both major pathological events in the development of AD.

**Electronic supplementary material:**

The online version of this article (doi:10.1007/s00401-015-1395-2) contains supplementary material, which is available to authorized users.

## Introduction

Amyloid pathology and neuroinflammation including activation of glial cells are key hallmarks of the neuropathology in brains of Alzheimer’s disease (AD) patients. Both Abeta peptides and pro-inflammatory cytokines/chemokines are reported to interfere with neuronal survival and with proper synaptic function, resulting in cognitive decline [6, 20, 21, 62]. The relation between the actual clinical status of the patient and the degree of neuropathology can be assessed by testing cognitive function and by imaging techniques monitoring hippocampal shrinkage, Abeta deposition and microglial activation [26, 29, 55]. Abeta peptides are generated by proteolytical processing of the amyloid precursor protein (APP) and may undergo post-translational modification such as N-terminal truncation and subsequent cyclization of N-terminal glutamate (Glu) into pyroglutamate (pGlu) [43, 46, 47, 56]. The resulting pGlu-Abeta peptides (1) are major constituents of Abeta deposits in sporadic and familial AD [33, 40, 41, 46], (2) possess a high aggregation velocity [13, 18, 31, 44, 51, 54], (3) display resistance to degradation by peptidases [45] and (4) are particularly neurotoxic to primary neurons, neuronal cell lines and neurons of APP transgenic animals in vivo [1, 2, 38, 44, 61]. Interestingly, pGlu-modified Abeta peptides in brains of AD patients and transgenic mouse models were reported to be closely associated with [^11^C]Pittsburgh Compound-B (PIB) autoradiographic signals [28].

The pGlu-Abeta peptide modification has been demonstrated to be catalyzed by glutaminyl cyclase (QC) in vitro [50] and in vivo [11, 12, 49, 53]. Recently, we observed robust QC expression in mouse and human brain in AD-vulnerable subcortical regions such as nucleus basalis Meynert, locus coeruleus and Edinger–Westphal nucleus [35] and in a subpopulation of neocortical neurons and of GABAergic interneurons in hippocampus [15, 16]. Chronic pharmacological inhibition or genetic ablation of QC activity in animal models of AD resulted in reduced pGlu-Abeta peptide generation and in ameliorated behavioral deficits [2, 22, 53], while QC overexpression aggravated neuropathology and cognitive dysfunction in transgenic mice [22].

Recently, a Golgi-resident isoenzyme of QC with identical enzymatic characteristics was discovered [10, 59]. In cell-free assays, both enzymes convert a variety of substrates with similar kinetics and are inhibited by a number of inhibitors from different chemical classes at comparable *K*_i_ values [59]. However, based on the cell type-specific expression and subcellular localization, QC and isoQC appear to have distinct physiological substrate profiles. For example, isoQC—but not QC—catalyzes the pGlu modification of the monocyte chemoattractant protein-1 (MCP-1; also designated CCL2) and thereby stimulates monocytic infiltration into the peritoneum of mice in the thioglycollate-induced peritonitis model [8]. CCL2 lacking the pGlu modification has reduced chemoattractant activity and is less resistant to proteolytical degradation by aminopeptidases [8]. Recent studies demonstrated that isoQC-catalyzed pGlu-CCL2 also contributes to inflammatory tissue damage in mouse models of septic arthritis and non-alcoholic fatty liver disease [9, 19]. Since the tissue damage is less severe in isoQC knock-out mice and has been shown to be ameliorated by isoQC inhibitors, it points towards a role of isoQC in chronic inflammatory processes.

In AD, CCL2 was also shown to play a dominant role in chronic inflammation [58] and to be associated with a faster rate of cognitive decline at early stages of the disease [60]. Likewise, CCL2 overexpression accelerated Abeta oligomer and diffuse plaque formation and caused deficits in spatial and working memory in mouse models of AD [25, 63], while expression of a dominant negative CCL2 variant ameliorated Abeta pathology and reduced memory impairments [24]. Chronic neuroinflammation triggered by a variety of stimuli including—but not limited to—Abeta peptide aggregates was shown to contribute to cognitive decline in AD patients [32, 42].

So far, the enzymatic mechanisms of pGlu-CCL2 formation in brain, the brain region and cell type-specific expression pattern of isoQC, its subcellular localization and correlation with Abeta pathology in brains of transgenic mice and AD patients are not yet known and were therefore the subject of this study. Collectively, we here report for the first time a role for isoQC in CCL2 maturation and bioactivity in brain which may contribute to aspects of AD pathology and to the manifestation of clinical symptoms.

## Materials and methods

### Mouse brain tissue

Brain tissue of wild-type and APP transgenic Tg2576 mice at the postnatal age of 4 and 17 months was used for immunohistochemical, ELISA and quantitative reverse transcriptase PCR (qRT-PCR) analyses. IsoQC knock-out mice described in Cynis et al. [8] served as negative control for the specificity of the isoQC antibodies employed.

For immunohistochemistry, mice were anesthetized with pentobarbital and transcardially perfused with 25 ml of phosphate-buffered saline (PBS, 0.01 M; pH 7.4) followed by perfusion with 25 ml of 4 % paraformaldehyde in phosphate buffer (PB, 0.1 M; pH 7.4). Brains were removed from the skull and post-fixed by immersion in the same fixative overnight at 4 °C. After cryoprotection in 30 % sucrose in 0.1 M PB for 3 days, brains were snap-frozen in *n*-hexane at −68 °C and stored at −20 °C. Coronal sections (30 µm) were cut on a sliding microtome and collected in 0.1 M PB.

For biochemical analyses, mice were sacrificed by decapitation at the postnatal age indicated, brains were removed from the skull, and neocortex was prepared, snap-frozen in liquid nitrogen and stored at −80 °C pending qRT-PCR or ELISA analyses.

### isoQC and CCL2 antibodies

**Fig. 1:**
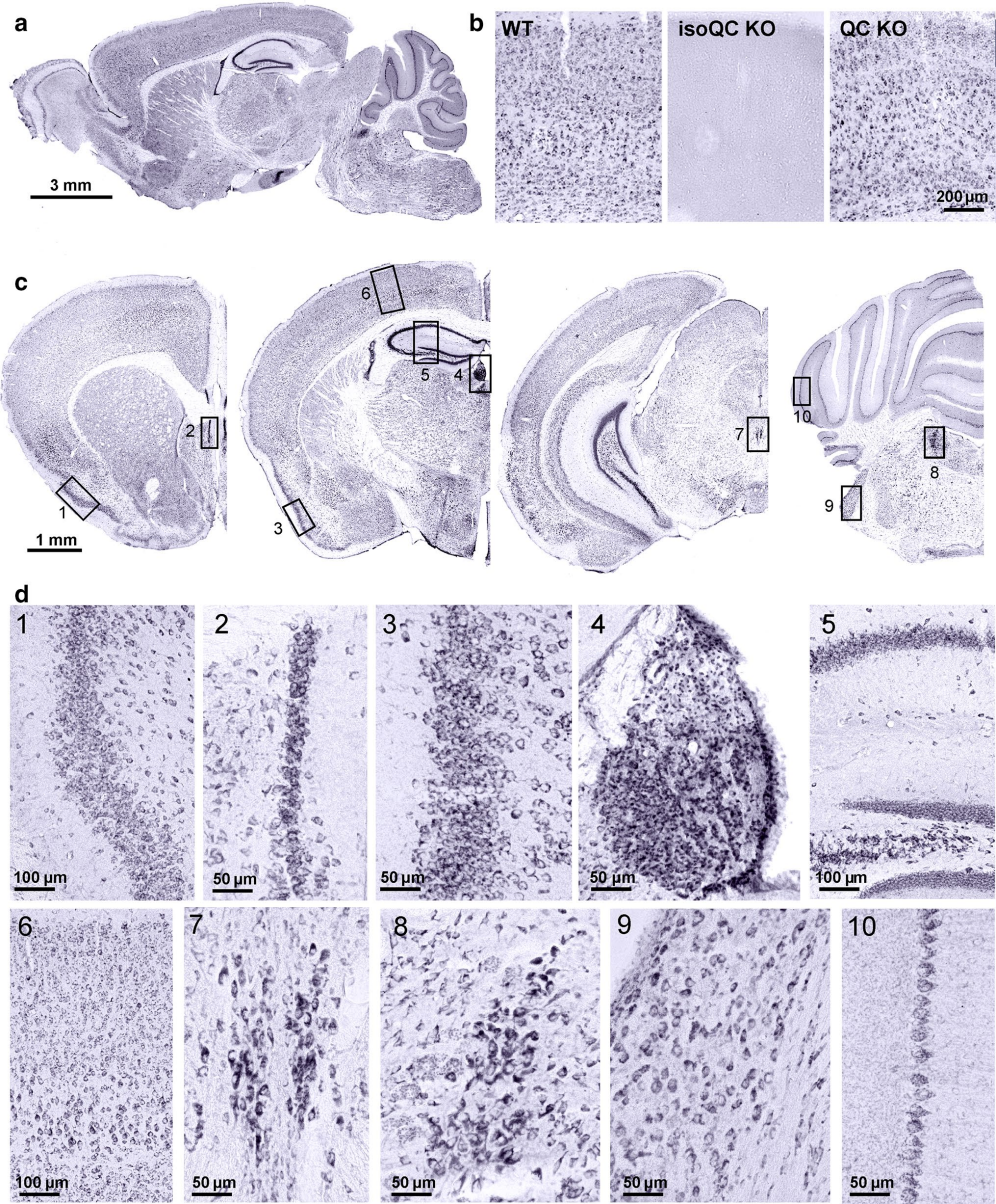
Expression of isoQC in mouse brain. **a** The enzyme isoQC is expressed almost ubiquitously in numerous neocortical, hippocampal, subcortical and cerebellar brain structures of wild-type mouse brain at 4 months of age as evidenced by immunohistochemical labeling in sagittal sections. **b** This labeling is highly specific as shown by the absence of staining in brain tissue of isoQC knock-out mice. On the other hand, in QC knock-out brain tissue the isoQC labeling is not affected. **c** In coronal sections, some brain regions with particularly high expression levels of isoQC can be distinguished. These include piriform *1* entorhinal *3* and pyramidal layer V *6* cortices, hippocampal structures such as indusium griseum *2*, Cornu ammonis 1–4 and dentate gyrus *5*, as well as habenular *4* and Edinger–Westphal *7* nuclei, locus coeruleus *8*, cochlear nucleus *9* and Purkinje cells of the cerebellum *10*. **d** At higher magnification labeled cells in all these brain regions appear to have a neuronal shape and size. *1* piriform cortex*, 2* indusium griseum*, 3* entorhinal cortex*, 4* habenular nucleus*, 5* hippocampus*, 6* parietal cortex*, 7* Edinger–Westphal ncl., *8* locus coeruleus, *9* cochlear nucleus, *10* Purkinje cells

Since the specificity of the immunohistochemical isoQC labeling is critical for this study, we tested a number of isoQC antibodies in brain tissue of wild-type, QC knock-out and isoQC knock-out mice. The antiserum 3285 (Probiodrug, Halle/S., Germany) allowed for the specific immunohistochemical detection of isoQC in mouse brain. This staining was completely absent in brains of isoQC knock-out mice, but not in brains of QC knock-out mice (Fig. 1).

Additionally, different anti-CCL2 antibodies derived from mouse and goat, respectively, were tested. The goat anti-CCL2 antiserum sc-1784 (St. Cruz) and the mouse anti-CCL2 antibody (clone 4B8; Probiodrug, Halle/Saale, Germany) showed a distinct staining pattern for CCL2 around nuclei of mouse primary neurons as well as of neurons in mouse brain tissue. The other tested antibodies displayed more diffuse and undifferentiated staining of brain sections or unspecific labeling of blood capillaries. In human brain tissue, the goat antiserum sc-1784 and the mouse monoclonal antibody MAB2791 (R&D Systems) demonstrated distinct neuronal CCL2 labeling comparable to the staining pattern observed in mouse brain.

Based on specificity and the superior signal-to-background ratio, the rabbit anti-isoQC antiserum 3285 and the goat anti-CCL2 antiserum sc-1784 were selected for immunohistochemistry in mouse brain tissue and the rabbit anti-isoQC antiserum 3285 as well as the monoclonal anti-CCL2 antibody MAB2791 was used in human brain tissue.

### isoQC immunohistochemistry in mouse brain

Immunohistochemistry to detect isoQC was performed using the affinity-purified rabbit antiserum 3285 at a dilution of 1:500. After inactivation of endogenous peroxidase with 0.6 % H_2_O_2_ in 0.1 M TBS for 15 min and blocking of unspecific binding sites with 5 % normal goat serum in TBS containing 0.3 % Triton-X100, brain sections were incubated in the same solution with the primary antibody at 4 °C overnight in a humid chamber on a shaker. The following day, sections were incubated with secondary biotinylated goat anti-rabbit antibodies (Dianova; 1:400) in TBS-2 % bovine serum albumin (BSA) for 60 min at room temperature followed by the ABC method which comprised incubation with complexed streptavidin-biotinylated horseradish peroxidase (Invitrogen). Incubations were separated by washing steps (3 times 5 min in TBS). The isoQC immunoreaction was visualized by incubation with 4 mg 3,3′-diaminobenzidine (DAB) and 2.5 µl H_2_O_2_ per 5 ml Tris buffer (0.05 M, pH 7.6) for 1–2 min.

### Double immunofluorescent labeling procedures

To reveal (1) the cell type-specific expression, (2) the intracellular localization and (3) the co-localization of isoQC with its putative substrate CCL2, the rabbit anti-isoQC antiserum 3285 was used in combination with mouse monoclonal antibodies or a polyclonal antiserum from goat or chicken directed against neuron-, astrocyte- and compartment-specific marker proteins and against CCL2, respectively (see Table 1). Brain sections were incubated with cocktails of primary antibodies overnight at 4 °C. On the next day, sections were washed three times with TBS and were then incubated with biotinylated donkey anti-rabbit, 1:400 and Cy3-conjugated donkey anti-mouse, -goat and -chicken, 1:200 (Dianova) secondary antibodies, respectively, followed by Cy2-conjugated streptavidin 1:100 (Dianova) for 60 min at room temperature. After mounting the brain sections on microscope slides, cellular nuclei were counterstained with ToPro3 (1:10,000; Life Technologies).

**Table 1:** Cocktails of antibodies used for double and triple labeling immunohistochemistry

Primary antibody	Dilution	Host	Company	Secondary antibody
isoQC 3285 HuC/D	1:100 1:100	Rabbit Mouse	Probiodrug Invitrogen	Bio-donkey anti-rabbit + SA-Cy2 Cy3 donkey anti-mouse
isoQC 3285 GFAP	1:100 1:100	Rabbit Goat	Probiodrug Santa Cruz	Bio-donkey anti-rabbit + SA-Cy2 Cy3 donkey anti-goat
isoQC 3285 WGA Alexa555	1:100 1:500	Rabbit Lectin	Probiodrug Invitrogen	Bio-donkey anti-rabbit + SA-Cy2
isoQC 3285 CCL2 sc-1784	1:100 1:100	Rabbit Goat	Probiodrug Santa Cruz	Bio-donkey anti-rabbit + SA-Cy2 Cy3 donkey anti-goat
isoQC 3285 calreticulin	1:100 1:200	Rabbit Chicken	Probiodrug Abcam	Bio-donkey anti-rabbit + SA-Cy2 Cy3 donkey anti-chicken
isoQC 3285 syntaxin-6	1:100 1:100	Rabbit Mouse	Probiodrug Abcam	Bio-donkey anti-rabbit + SA-Cy2 Cy3 donkey anti-mouse
CCL2 sc-1784 HuC/D	1:100 1:100	Goat Mouse	Santa Cruz Invitrogen	Bio-donkey anti-goat + SA-Cy2 Cy3 donkey anti-mouse
CCL2 sc-1784 GFAP-Cy3	1:100 1:1,000	Goat Mouse	Santa Cruz Sigma	Bio-donkey anti-goat + SA-Cy2
CCL2 MAB2791 GFAP	1:1,000 1:100	Mouse Goat	R&D Systems Santa Cruz	Bio-donkey anti-mouse + SA-Cy2 Cy3 donkey anti-goat
CCL2 sc-1784 Iba-1	1:100 1:100	Goat Rabbit	Santa Cruz Wako	Bio-donkey anti-goat + SA-Cy2 Cy3 donkey anti-rabbit
ToPro	1:10,000		Invitrogen	
isoQC 3285 CCL2 sc-1784 GFAP	1:100 1:100 1:1,000	Rabbit Goat Mouse	Probiodrug Santa Cruz Sigma	Bio-donkey anti-rabbit + SA-Cy2 Cy3 donkey anti-goat Cy5 donkey anti-mouse
isoQC 3285 CCL2 sc-1784 pGlu-Abeta	1:100 1:100 1:100	Rabbit Goat Mouse	Probiodrug Santa Cruz Synaptic Systems	Bio-donkey anti-rabbit + SA-Cy2 Cy3 donkey anti-goat Cy5 donkey anti-mouse

Secondary antibodies were all from Dianova and used at a dilution of 1:400 (biotinylated) and 1:200 (fluorochromated)

### Confocal laser scanning microscopy

Confocal laser scanning microscopy (LSM 510, Zeiss, Oberkochen, Germany) was performed to reveal co-localization of isoQC and intracellular compartment markers as well as with CCL2. For Cy2-labeled antigens (green fluorescence), an argon laser with 488 nm excitation and 510 nm emission wavelength was used applying a low-range band pass (505–530 nm). For Cy3-labeled antigens, a helium–neon laser with 543 nm excitation and 570 nm emission was used applying high-range band pass (560–615 nm). The nuclear staining compound ToPro3 was visualized by excitation at 642 nm and emission at 661 nm.

### Mouse primary neuronal and astrocyte cultures

Primary neuronal cell cultures were derived from fetal mouse brain at gestation day 16 as described by Löffner et al. [27]. After 3 days in vitro, cultures were treated with 10 µM cytosine-1-β-d-arabinofuranoside to suppress the growth of dividing cells, mainly of astroglial origin. Astrocyte-rich primary cell cultures were derived from brains of newborn mice according to Löffner et al. [27] and were maintained in DMEM-based medium. Cells were cultured at 37 °C in a humidified atmosphere with 95 % air/5 % CO_2_ and the medium was renewed every 1–3 days. After 7 days in culture, astrocytes were incubated in Abeta (5 µM), pGlu-Abeta (5 µM), LPS (1 µg/ml medium; O55:B5, Sigma), IFN-γ (20 ng/ml medium; Sigma) or a combination of LPS/IFN-γ for 12, 24, 48 and 72 h, respectively. Abeta peptides were synthesized in 50 µmol scale on Fmoc-Val/Ala-NovaSyn-TGA resin (0.15 mmol/g) using an automated Symphony Synthesizer (Rainin) as described in detail in [51]. The crude peptides were dissolved in HFIP for preparative HPLC and further purified by RP-HPLC [51]. Peptide purity and identity were confirmed by analytical HPLC (150 × 4.6, 5 µ Source or Gemini) and matrix-assisted laser desorption ionization mass spectroscopy (MALDI-MS). Prior to use, the peptides were dissolved in 1 mM HFIP and incubated at room temperature for 2 h to solubilise peptides and to monomerise β-sheet protein aggregates. Following evaporation of HFIP the peptides were dissolved in Optimen medium and diluted to the final concentration indicated.

### Human brain tissue

#### Case recruitment and characterization of human brain tissue

Case recruitment and autopsy were performed in accordance with guidelines effective at Banner Sun Health Research Institute Brain Donation Program of Sun City, AZ, USA [4]. The required consent was obtained for all cases. The definite diagnosis of AD for all cases used in this study was based on the presence of neurofibrillary tangles and neuritic plaques in the hippocampal formation and neocortical areas and met the criteria of the National Institute on Aging (NIA) and the Consortium to establish a registry for AD (CERAD) [34]. Brain tissue of temporal cortex (Brodmann Area 22) from 6 controls and 6 age-matched AD cases was used for isoQC, CCL2, GFAP and pGlu-Abeta immunohistochemistry. Additionally, for biochemical analyses, temporal cortex (Brodmann Area 22) of 7 control cases and 5 AD cases with a very short postmortem interval of 1.5–5.5 h and a thorough clinical characterization was used (Table 2). Anatomical structures and cortical layers were identified using consecutive Nissl-stained sections and the atlas of the human brain [30].

**Table 2:** Human brain tissue used for biochemistry and correlation analyses

	Case #	PMD (h)	Gender	Age (years)	Brain weight (g)	COD	Braak score	MMSE (last score)	CERAD Neuritic Plaque Score	CERAD Criteria	NIA criteria (likelihood of dementia)
Control	Co1	2.5	Female	86	1,145	Pulmonary fibrosis	III	27/30	0	Not met	Not met
	Co2	2.5	Female	87	1,120	Pneumonia/cancer	IV	30/30	0	Not met	Not met
	Co3	2.75	Female	75	1,110	Pulmonary embolus	III	29/30	0	Not met	Not met
	Co4	3.0	Male	82	1,240	Renal failure/COPD	III	26/30	A	Not met	Not met
	Co5	1.66	Male	78	1,460	Lung cancer/heart failure	I	28/30	0	Not met	Not met
	Co6	5.5	Male	89	1,275	Respiratory distress/COPD	II	30/30	0	Not met	Not met
	Co7	4.75	Male	86	1,400	Cardiac arrest	I	28/30	0	Not met	Not met
Mean		3.25	3/4	83.3	1,250						
AD	AD1	1.83	Female	84	1,080	End-stage AD	VI	0/30	C	Definite AD	High
	AD2	1.5	Female	85	940	Breast cancer/end-stage AD	VI	2/30	C	Definite AD	High
	AD3	3.16	Male	80	1,000	End-stage AD	VI	1/30	C	Definite AD	High
	AD4	2.25	Male	78	1,120	End-stage AD	VI	7/30	C	Definite AD	High
	AD5	2.5	Male	87	1,100	End-stage AD	VI	13/30	C	Definite AD	High
Mean		2.25	2/3	82.8	1,048						

*AD* Alzheimer’s disease, *PMD* postmortem delay, *Co* control, *COD* cause of death, *MMSE* Mini-Mental State Examination, *CERAD* Consortium to Establish a Registry for Alzheimer’s Disease, *NIA* National Institute on Aging

#### Tissue preparation

For immunohistochemistry, 15-mm-thick tissue blocks were prepared in the frontal plane according to the atlas of the human brain [30] and were fixed in 4 % paraformaldehyde in 0.1 M PBS, pH 7.4 for 3–4 days. Areas containing the regions of interest were cryoprotected in 30 % sucrose in 0.1 M PBS, pH 7.4. Series of 30-µm-thick sections were cut on a freezing microtome and collected in PBS containing 0.1 % sodium azide.

For biochemical analyses including Abeta and CCL2 ELISAs, and for quantitative RT-PCR to detect isoQC and CCL2 transcripts, unfixed temporal cortex tissue with a short postmortem interval was stored at −80 °C (see Table 2). Brain tissue (10 % w/v) was homogenized in TBS (20 mM Tris, 137 mM NaCl, pH 7.6) containing protease inhibitor cocktail (Complete Mini, Roche), sonicated and then centrifuged at 75,500×*g* for 1 h at 4 °C and the supernatant was stored at −80 °C. Abeta peptides were sequentially extracted with TBS/1 % Triton X-100 (TBS/triton fraction), 2 % SDS in distilled water (SDS fraction), and 70 % formic acid (FA fraction). The combined SDS and FA fractions were considered as the insoluble pool of Abeta. For determination of CCL2, brain tissue was homogenized (Precellys homogenizer, Peqlab) in the threefold volume (w/v) of ice-cold ELISA blocker (Thermo Scientific) containing protease inhibitors. The homogenate was centrifuged (10 min, 15,000×*g*), the supernatant removed and subjected to another centrifugation step (30 min, 25,000×*g*). The final supernatant was shock-frozen and stored at −80 °C.

### Immunohistochemistry of human brain tissue

#### Nissl staining

Coronal sections of the human hippocampus were mounted on gelatin-coated slides and stained in 0.1 % cresyl violet according to standard protocols.

#### Single labeling isoQC and CCL2 immunohistochemistry

All immunohistochemical procedures were performed on free-floating brain sections. Immunohistochemistry to detect isoQC and CCL2 in human brain was performed using the rabbit anti-isoQC antiserum 3285 (1:500) and the mouse monoclonal CCL2 antibody MAB2791 (1:500), respectively. Brain sections were treated with 2 % H_2_O_2_ in 60 % methanol for 1 h, to abolish endogenous peroxidase activity. Unspecific staining was blocked in PBS-T containing 2 % BSA, 0.3 % milk powder and 0.5 % normal donkey serum before incubating brain sections with the primary antibodies at 4 °C overnight. The following day, sections were incubated with secondary biotinylated donkey anti-rabbit or donkey anti-mouse antibodies (Dianova; 1:1,000) for 60 min at room temperature followed by the ABC method which comprised incubation with complexed streptavidin-biotinylated horseradish peroxidase. Incubations were separated by washing steps (3 times 5 min in PBS-T). Binding of peroxidase was visualized by incubation with 2 mg DAB, 20 mg nickel ammonium sulfate and 2.5 µl H_2_O_2_ per 5 ml Tris buffer (0.05 M; pH 8.0) for 1–2 min, resulting in black labeling.

#### Double and triple labeling immunohistochemistry

Simultaneous immunohistochemical double and triple labeling of isoQC and its putative substrate CCL2 in combination with the astrocyte marker GFAP or with pGlu-Abeta were performed by incubations of brain slices in cocktails of primary antibodies from different species (Table 1) at 4 °C overnight following the procedure for mouse brain described above.

### qRT-PCR for isoQC and CCL2

Mouse and human brain tissue samples and cultured primary mouse astrocytes were homogenized using a Precellys homogenizer with 1.4-mm ceramic beads (5,000 rpm, 30 s, Peqlab). RNA was isolated using the NucleoSpin RNA II kit (Macherey–Nagel) according to the manufacturer’s instructions. RNA concentration was measured using a NanoDrop 2000 spectrophotometer (Peqlab). RNA (0.1 µg) was reverse transcribed to cDNA using random primers (Roche) and Superscript III (Life Technologies). Quantitative real-time PCR was performed in a Rotorgene3000 (Corbett Research) using the Rotor-Gene SYBR Green PCR kit and the Quantitect primer assay HsQPCTL (Qiagen), or specific primers for HsCCL2, MsCCL2 and MsQPCTL synthesized by Metabion (Martinsried, Germany) [8, 23]. Relative amounts of gene expression were determined with the Rotorgene software version 6.1 in comparative quantitation mode. Normalization was done against the most stably expressed reference gene YWHAZ identified using Normfinder [3]. The PCR was verified by product melting curves and single amplicons were confirmed by agarose gel electrophoresis.

### Abeta and CCL2 ELISAs

Specific ELISAs to detect Abeta x-42 and pGlu-Abeta3-42 (IBL, Hamburg) in human brain were performed as described by Schilling et al. [53]. CCL2 concentrations in mouse and human brain were quantified by ELISA (Thermo Scientific) as described earlier [8]. All samples were analyzed in triplicate and the concentrations of the respective Abeta peptides present in temporal cortex were calculated from a standard curve.

### IsoQC Western blot analyses

To analyze the amount of isoQC protein in mouse brain, immunoblotting was carried out. In initial experiments, four different anti-isoQC antibodies were tested for their specificity and sensitivity in brain tissue of wild-type, QC knock-out and isoQC knock-out mice. The antiserum 5407 (Probiodrug, Halle/S., Germany) generated a specific band at the molecular weight of isoQC (43 kDa) when testing homogenates of wild-type and QC knock-out mice, but not isoQC knock-out mice (not shown). Tissue homogenates from cortex of wild-type and Tg2576 mice were loaded on a 12 % SDS polyacrylamide gel, separated by electrophoresis and transferred onto polyvinylidene difluoride membranes. The membranes were blocked overnight at 4 °C in TBS-T, pH 7.4, and 4 % BSA and subsequently incubated with primary rabbit anti-isoQC antibody 5407 (1:750) and with a secondary peroxidase-conjugated goat anti-rabbit antibody (1:1,000) for 1 h each. After washing the membranes, the bound proteins were visualized by a luminol detection system (Santa Cruz). Normalization of isoQC expression was achieved after stripping and incubation in a mouse anti-β-actin antibody, 1:16,000 and subsequent detection by a secondary POD-conjugated donkey anti-mouse antibody (1:10,000), followed by visualization with luminol detection system as described above. The blots were digitalized by the software GelCapture and the images were evaluated by densitometric image analysis using the software package TINA 2.0 (RAYTEST, Straubenhardt, Germany). The sum of gray values over each individual band obtained by densitometry was corrected for background and then the corrected values of the isoQC bands were compared to the corrected values of the β-actin bands.

### Statistical analyses

Statistical analyses of qRT-PCR, ELISA and Western blot data were done by one-way ANOVA followed by Bonferroni post hoc test. Statistically significant differences are given for *p* values of <0.05 (*), <0.01 (**) and <0.001 (***) calculated using the unpaired *t* test with Welch’s correction. Correlation analyses of mRNA, ELISA and MMSE data were performed by calculating Pearson’s correlation coefficient *r* with GraphPad Prism4 software.

## Results

### IsoQC expression in mouse brain and co-localization with CCL2

In sagittal brain sections of 4-month-old mice, widespread isoQC immunoreactivity was observed in numerous brain regions from olfactory bulb to cerebellum and spinal cord (Fig. 1a). To determine the specificity of this unexpectedly abundant isoQC labeling, brain tissue of wild-type, isoQC knock-out and QC knock-out mice was analyzed. The labeling generated by the isoQC antiserum 3285 in wild-type mouse brain was completely absent in isoQC knock-out but not in QC knock-out mouse brain tissue demonstrating the specificity of this antiserum for isoQC (Fig. 1b). In wild-type mouse brain, significant proportions of neurons in neocortical (cingulate, frontal, parietal, piriform, entorhinal cortex), hippocampal (indusium griseum, cornu ammonis 1–4, dentate gyrus) and subcortical (Edinger–Westphal nucleus, habenular and cochlear nucleus, locus coeruleus) brain structures were found to be isoQC-immunoreactive (Fig. 1c, d). The intensity of the isoQC labeling was strongest in hippocampal pyramidal, granular cells and indusium griseum, habenular, Edinger–Westphal nucleus, piriform and entorhinal cortex neurons (Fig. 1c, d).

**Fig. 2:**
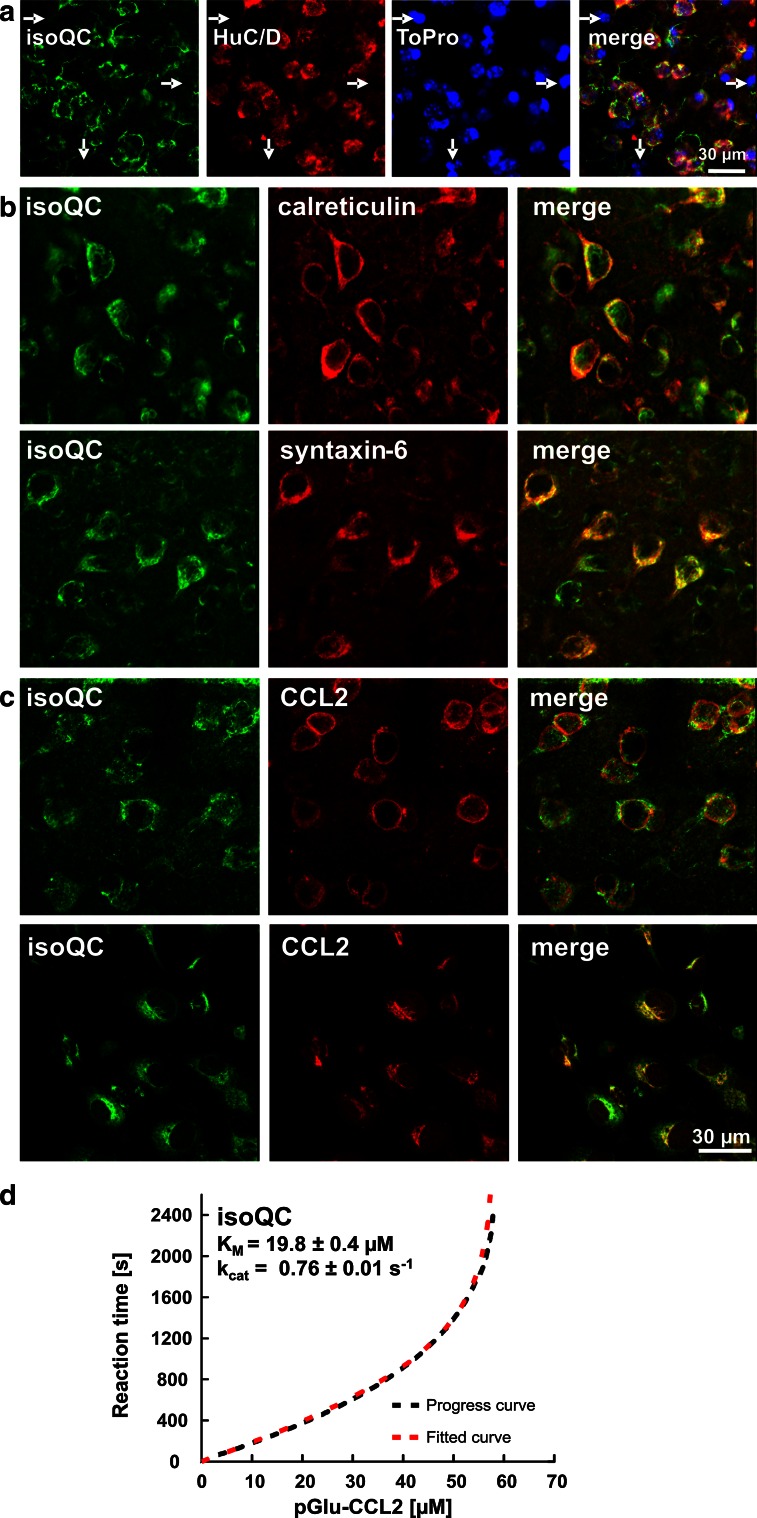
Neuronal isoQC expression, subcellular localization and co-expression of CCL2 in mouse brain and primary neurons. **a** IsoQC was strictly co-localized with the neuronal marker HuC/D in brain sections of 17-month-old wild-type mice demonstrating a neuron-specific expression. Cells which did not display HuC/D immunoreactivity were also negative for isoQC labeling (*arrows*). **b** IsoQC was co-localized with cellular compartment markers calreticulin and syntaxin-6, consistent with a subcellular localization in endoplasmic reticulum and Golgi apparatus. **c** The putative isoQC substrate CCL2 was found to be co-expressed by isoQC-immunoreactive neurons in cortex of wild-type mice (*top*) and in primary neuronal cultures (*bottom*). **d** The conversion of CCL2 by recombinant isoQC was analyzed in a kinetic assay. The progress curve (*black trace*) was in accordance with a curve modeled according to the integrated form of the Michaelis–Menten equation (*red trace*), enabling the determination of the kinetic parameters *K*_M_ (19.8 ± 0.4 μM) and *k*_cat_ (0.76 ± 0.01 s^−1^)

In the unmanipulated 4-month-old mouse brain, the expression of isoQC was restricted to neurons as shown by co-localization with the neuronal marker HuC/D and by the absence of isoQC labeling in HuC/D-negative cells (Fig. 2a). Intracellularly, isoQC was present in endoplasmic reticulum and Golgi structures as shown by co-localization with calreticulin and with syntaxin-6, respectively (Fig. 2b). Neurons immunoreactive for isoQC frequently co-expressed its putative substrate CCL2 in mouse brain and in mouse primary neurons (Fig. 2c). The conversion of Gln-CCL2 by recombinant isoQC was analyzed applying a kinetic assay that is based on consumption of NADH in a coupled reaction [52] (Fig. 2d). The progress curve was in accordance with a curve modeled according to the integrated form of the Michaelis–Menten equation, enabling the determination of the kinetic parameters *K*_M_ and *k*_cat._ The kinetic data show that CCL2 is converted by isoQC, but with lower efficiency compared to smaller peptide substrates. The observed co-localization of isoQC and CCL2 within intracellular compartments, i.e. at higher concentration of substrate and enzyme due to compartmentalization, might thus be important to facilitate the rather slow turnover of CCL2.

### Up-regulation of isoQC and CCL2 expression in APP transgenic Tg2576 mice

**Fig. 3:**
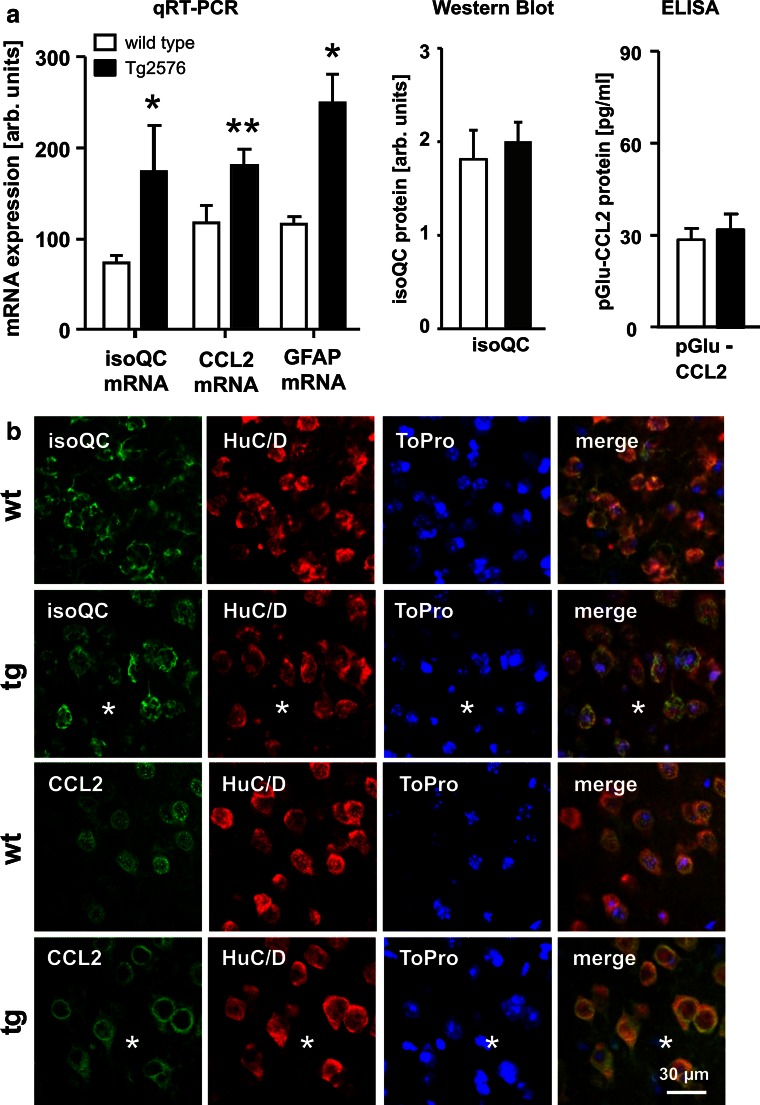

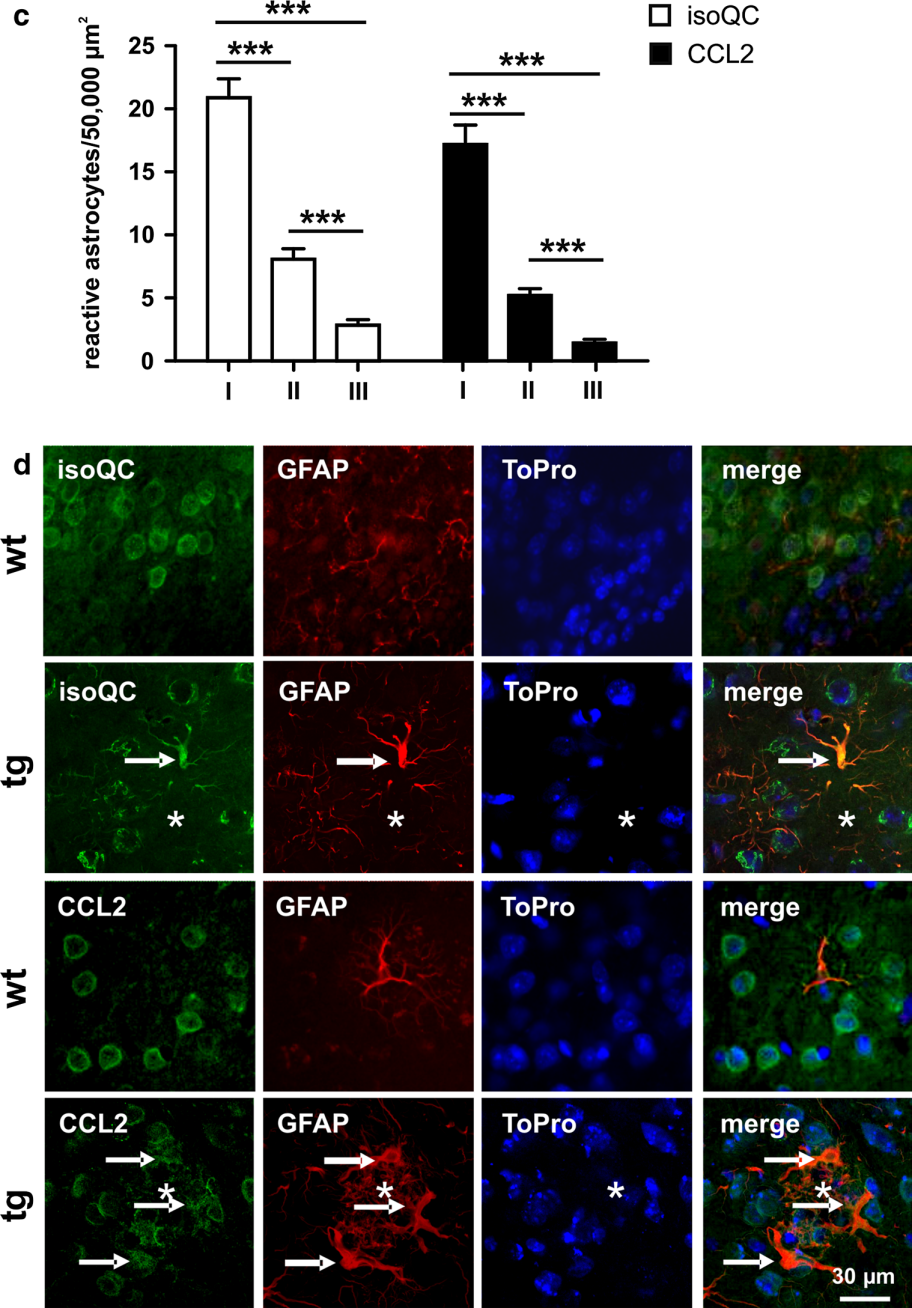
Regulation of isoQC and CCL2 expression in Tg2576 mice. **a** The neocortical isoQC and CCL2 mRNA levels were increased by 135 and 55 %, respectively, in 17-month-old APP transgenic Tg2576 mice (*black bars*) compared to wild-type littermates (*white bars*) as demonstrated by qRT-PCR analyses. Additionally, GFAP mRNA levels were increased by 115 %, indicating astrogliosis in Tg2576 mice. The isoQC and pGlu-CCL2 protein levels, however, were not affected in Tg2576 mice as measured by Western blot analysis and ELISA, respectively. **b** In 17-month-old wild-type (wt) and APP transgenic Tg2576 mice (tg) there was a predominantly neuronal expression of isoQC and CCL2 (*green* immunofluorescence) as revealed by co-expression of HuC/D (*red* immunofluorescence). The *asterisks* indicate the position of Abeta plaques in Tg2576 tissue. **c** In addition, aged Tg2576 mice—but not wild-type littermates—displayed astrocytic expression of isoQC and CCL2 in proximity of Abeta deposits following a gradient from the core towards the periphery of plaques (*I*, within plaque core diameter; *II*, double plaque core diameter; *III*, triple plaque core diameter). **d** The astrocytic co-expression (*arrows*) of isoQC and CCL2 in proximity of Abeta plaques (*asterisks*) is visualized by the co-expression of the astrocyte marker GFAP (*red* immunofluorescence). *p < 0.05, **p < 0.01, ***p < 0.001

To reveal a possible effect of transgenic human APP expression and Abeta plaque formation on the expression of isoQC and its putative substrate CCL2, both transcripts were quantified in neocortex of 17-month-old wild-type and APP transgenic Tg2576 mice. The isoQC mRNA levels were increased by a factor of 2.35 in neocortex of Tg2576 mice compared to wild-type littermates (Fig. 3a). A statistically significant up-regulation by 55 % was also observed for CCL2 transcript levels in brains of Tg2576 mice, indicating a pro-inflammatory response (Fig. 3a). The qRT-PCR data were validated by the demonstration of an up-regulation of GFAP mRNA, a well-known astrocytic response to Abeta plaque pathology (Fig. 3a).

The isoQC and pGlu-CCL2 protein levels quantified by Western blot analysis and ELISA, however, were not significantly increased in cortex of Tg2576 mice compared to wild-type littermates (Fig. 3a). This is also illustrated by similar neuronal immunofluorescent labeling intensity for isoQC and for CCL2 in cortex of wild-type compared to Tg2576 mice (Fig. 3b).

However, with regard to the cell type-specific isoQC and CCL2 expression, we observed remarkable differences between aged wild-type and Tg2576 mice. While isoQC and CCL2 were neuron-specifically expressed in wild-type mice, both proteins were additionally induced in neocortical reactive astrocytes in proximity of Abeta plaques of 17-month-old Tg2576 mice (Fig. 3c, d). There was a gradient in the number of isoQC- and CCL2-immunoreactive astrocytes per area from the plaque core towards the plaque periphery ranging from 21 isoQC- and 17 CCL2-immunoreactive astrocytes, respectively, per 50,000 µm^2^ in the core to 3 isoQC- and 2 CCL2-immunoreactive astrocytes per 50,000 µm^2^ in the periphery (Fig. 3c). This indicates an Abeta-dependent mechanism of the induction of both proteins.

### Simultaneous induction of isoQC and CCL2 in mouse primary astrocytes

Next, we were interested in establishing a cellular model that allows investigating the regulation of isoQC and CCL2 in astrocytes. Therefore, mouse primary astrocytes were cultured and the mRNA and protein levels of isoQC and CCL2 were analyzed under control conditions and after stimulation with Abeta peptides, to mimic a pathogenic situation present at the sites of Abeta deposits in brains of Tg2576 mice and AD patients. Additionally, to reveal whether a general, non-AD-related inflammatory stimulation induces astrocytic isoQC and CCL2 expression, astrocytes were also treated with LPS, IFN-γ, as well as a combination of both substances, for different time periods.

**Fig. 4:**
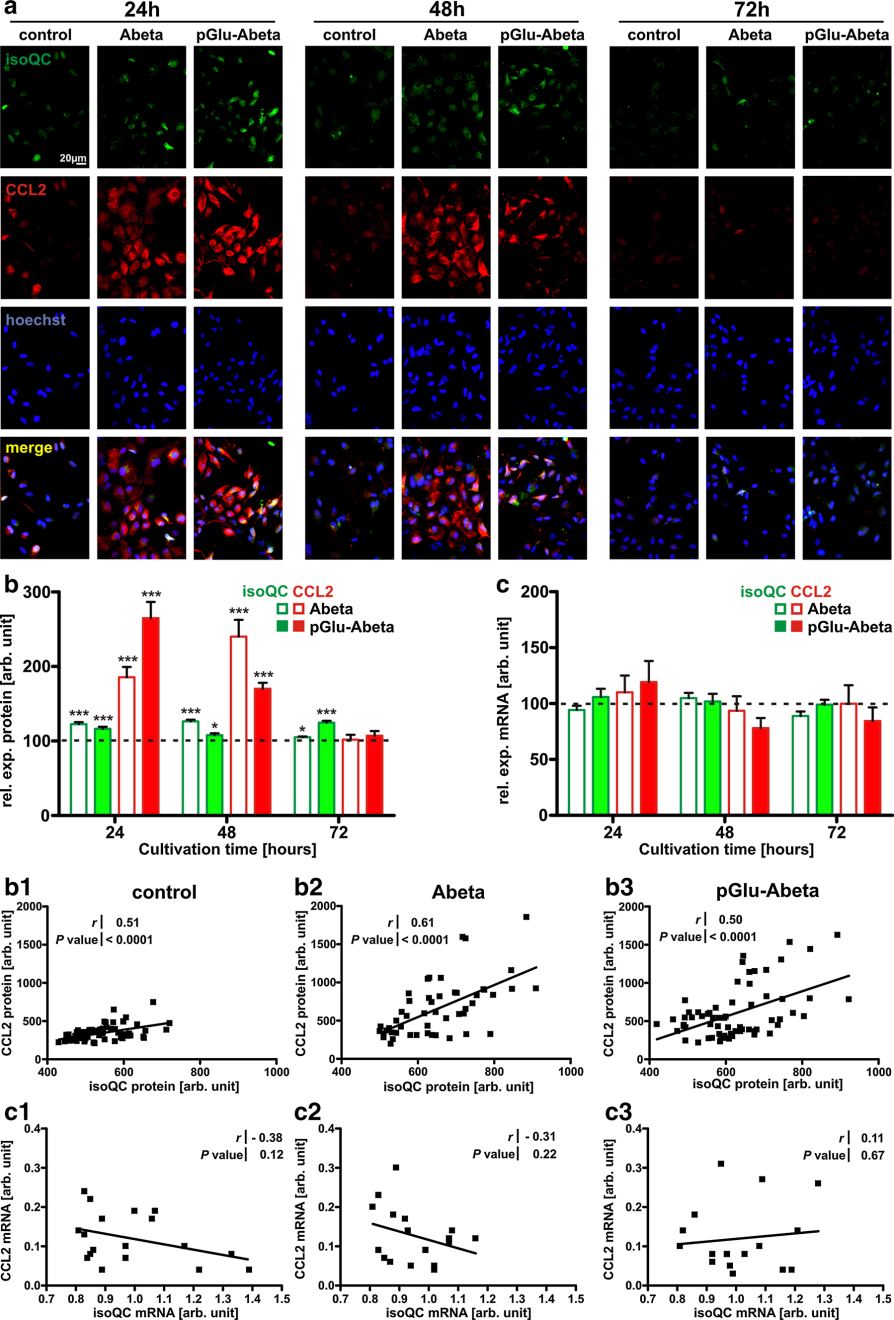
Co-regulation of isoQC and CCL2 in mouse primary astrocytes upon Abeta stimulation. **a** Immunocytochemical double labeling of isoQC (*green*) and CCL2 (*red*) with nuclear Hoechst counterstaining (*blue*) under control conditions and after stimulation with Abeta (5 μM) or pGlu-Abeta (5 μM) as indicated. Note the robust increase in the immunocytochemical labeling intensity for both proteins at 24 and 48 h and the decline at 72 h. **b** Quantification of immunocytochemical labeling revealed a highly significant time- and Abeta peptide-specific increase in isoQC and CCL2 immunoreactivity. Correlation analyses between isoQC and CCL2 immunocytochemical labeling demonstrated highly significant correlations in the expression of enzyme and substrate under control conditions (**b1**), after stimulation with Abeta (**b2**) and with pGlu-Abeta (**b3**). **c** Quantification of isoQC and CCL2 mRNA expression under control conditions and after stimulation with Abeta and pGlu-Abeta for different periods of time as indicated (*N* = 6 per time point). Note the absence of an increase of isoQC or CCL2 mRNA transcripts independent of the type and duration of the treatment. Correlation analyses of CCL2 mRNA levels plotted versus isoQC mRNA levels in individual astrocyte culture wells under control conditions and after (pGlu)-Abeta stimulation including all time points analyzed. Note the absence of a correlation between enzyme and substrate mRNA expression under control (**c1**) and treatment conditions (**c2**,** c3**)

While only weak immunocytochemical signals for isoQC and CCL2 protein were detected in untreated astrocytes, both proteins were transiently induced upon Abeta and pGlu-Abeta stimulation (Fig. 4a). CCL2 was maximally increased to 264 % (at 24 h of pGlu-Abeta stimulation) and to 240 % (at 48 h of Abeta stimulation), respectively, and declined to untreated control levels at 72 h after the onset of (pGlu)-Abeta treatment (Fig. 4b). The statistically significant induction of isoQC immunoreactivity was less pronounced after treatment with both Abeta variants but already present at 24 h and persisted until 72 h of treatment (Fig. 4b). Correlation analyses between expression levels of isoQC and CCL2 in untreated cells (Fig. 4b1), Abeta-stimulated cells (Fig. 4b2) and pGlu-Abeta-stimulated cells (Fig. 4b3) revealed a co-regulation of both proteins under all experimental conditions. In these analyses, data from all time points of one type of treatment were pooled.

The increased isoQC and CCL2 protein levels detected are not based on elevated expression of the respective mRNAs as demonstrated by qRT-PCR (Fig. 4c). There were no correlations between isoQC and CCL2 transcript levels for any of the treatments performed (Fig. 4c1–3).

This is in contrast to findings following a general pro-inflammatory challenge (LPS/IFN-γ stimulation), which revealed co-regulation of both isoQC and CCL2 transcript and protein expression in mouse primary astrocytes (see Supplementary Information 1).

### IsoQC and CCL2 expression in human control and AD cortex

**Fig. 5:**
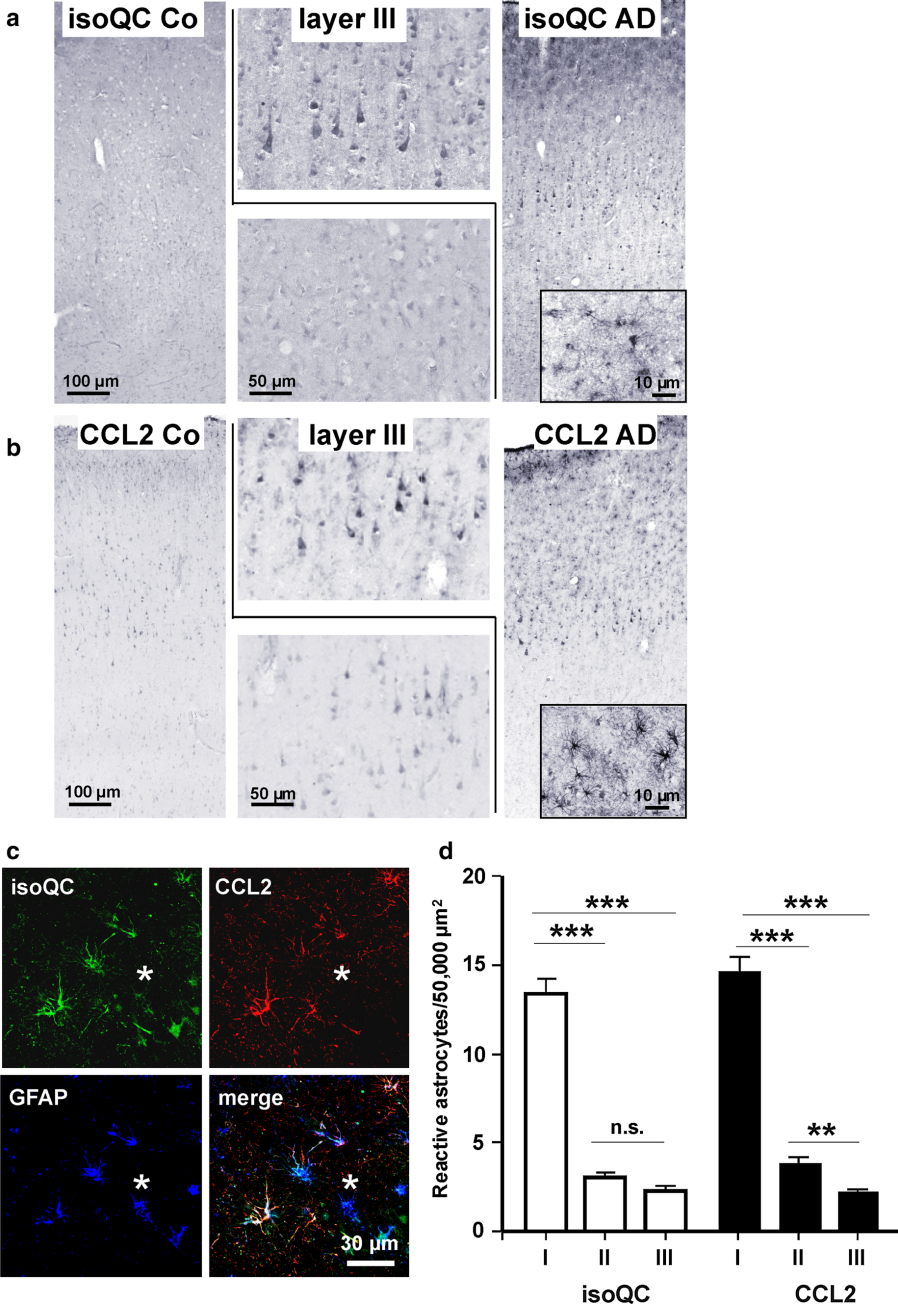
Immunohistological alterations of isoQC and CCL2 expression in AD cortex. **a** Immunohistochemistry for isoQC in human temporal cortex revealed a weak neuronal expression in control subjects. In corresponding tissue sections of AD subjects, an increased neuronal labeling intensity, in particular in layer III pyramidal neurons was observed. **b** A similar up-regulation in pyramidal layer III neurons was observed for CCL2. Additionally, both isoQC and CCL2 were induced in glia-like cells in proximity of Abeta plaques (**insets** in **a** and **b**). **c** Triple immunofluorescent labelings of isoQC with CCL2 and GFAP identified these glial cells as astrocytes surrounding Abeta deposits. **d** The astrocytic expression of isoQC and CCL2 in proximity of Abeta deposits followed a gradient from the core to the periphery of plaques (*I*, within plaque core diameter; *II*, double plaque core diameter; *III*, triple plaque core diameter). **p < 0.01, ***p < 0.001

To reveal a possible role of isoQC and CCL2 in AD, immunohistochemical labeling of human cortex from control and AD subjects was performed. In control tissue, a weak to moderate neuronal isoQC labeling across all cortical layers was observed (Fig. 5a). In contrast, a stronger neocortical isoQC labeling with the most robust staining intensity being present in layer III pyramidal neurons was detected in AD brain tissue (Fig. 5a). A similar labeling pattern and a higher staining intensity in layer III pyramidal neurons of AD subjects were also demonstrated for the isoQC substrate CCL2 (Fig. 5b).

In AD brain tissue, both isoQC and CCL2 immunoreactivity were detected in Abeta plaque-associated glial-like cells (insets in Fig. 5a, b). Based on the cellular morphology and on observations made in Tg2576 mouse brain (compare Fig. 3d), we assumed these glial cells to be most likely astrocytes. To validate this presumption, triple immunofluorescent labelings of isoQC, CCL2 and GFAP on well-preserved human brain tissue were performed. The resulting co-localization clearly demonstrated the co-induction of isoQC and CCL2 in Abeta plaque-associated reactive astrocytes (Fig. 5c) and supports the notion of an Abeta-driven inflammatory response involving isoQC-mediated pGlu-CCL2 formation. Quantitative analysis of plaque-related astrocytic isoQC and CCL2 expression revealed a steeply decreasing density of immunoreactive astrocytes (number of astrocytes per 50,000 µm^2^) from 13 isoQC- and 14 CCL2-expressing astrocytes per area in the plaque core, and 3 isoQC- and 4 CCL2-immunoreactive astrocytes in the adjacent rim around the plaque to 2 isoQC- and 2 CCL2-positive astrocytes in the outer rim most distant to the plaque, respectively (Fig. 5d). The Abeta plaque-associated expression of both proteins by reactive astrocytes was robust in 4 out of 6 and moderate in the remaining 2 AD cases. In controls, only one out of 6 cases displayed isoQC and CCL2 immunoreactivity associated with discrete Abeta deposits.

**Fig. 6:**
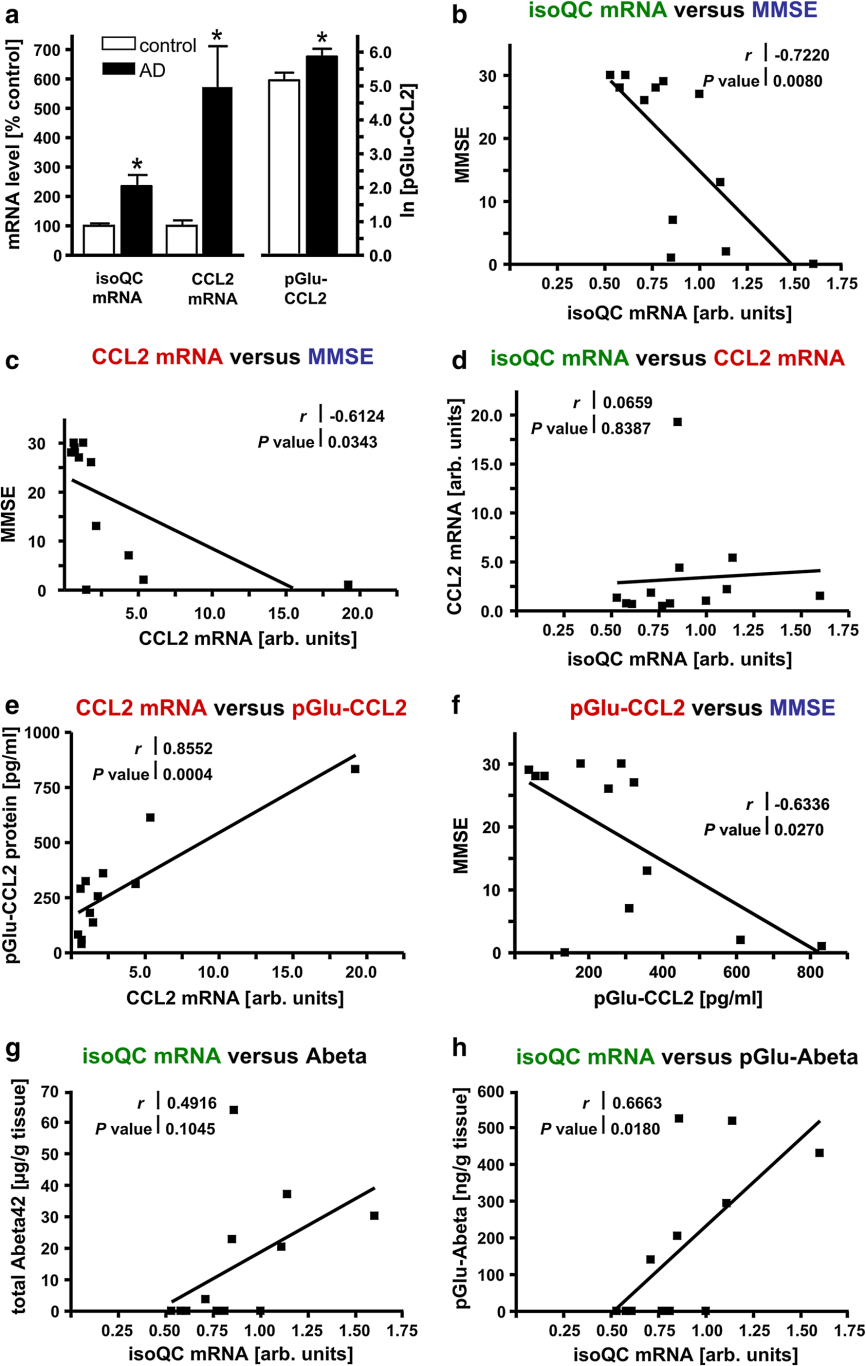
Characteristics of isoQC, CCL2 and pGlu-Abeta accumulation in AD and correlation analyses with MMSE. **a** Quantitative analyses revealed statistically significant increases of isoQC and CCL2 mRNA levels as well as pGlu-CCL2 protein in temporal cortex samples from AD cases compared to control subjects (*p < 0.05). **b** There was a statistically significant correlation between higher isoQC mRNA levels and the decline in MMSE (*r* = −0.7220; *p* = 0.0080). **c** Likewise, a statistically significant correlation between higher CCL2 mRNA levels and the decline in MMSE (*r* = −0.6124; *p* = 0.0343) was detected. **d** There was no correlation between isoQC and CCL2 transcript levels in the human brain samples analyzed. **e** A strong positive correlation between CCL2 mRNA and protein levels was detected (*r* = 0.8552; *p* = 0.0004). **f** As for CCL2 mRNA, an inverse correlation between pGlu-CCL2 protein and MMSE was revealed (*r* = −0.6336; *p* = 0.0270). While there was no correlation between isoQC mRNA levels and total Abeta42 peptide concentrations (**g**), a significant correlation between isoQC transcript levels and pGlu-Abeta concentrations was established (*r* = 0.6663; *p* = 0.0180) (**h**)

In the second set of human brain tissue samples, quantitative biochemical and molecular biological analyses were performed with the aim (1) to validate a possible role of isoQC in pGlu-Abeta and pGlu-CCL2 formation in human brain and (2) to analyze biochemical alterations such as expression of isoQC as well as pGlu-Abeta and pGlu-CCL2 formation with regard to a possible association with the clinical severity of AD as assessed by MMSE. To address this issue, pathologically and clinically well-characterized brain samples with a short postmortem interval were used (Table 2). By using qRT-PCR, an increase of isoQC mRNA levels to 235 ± 38 % and of CCL2 mRNA levels to 572 ± 139 % in AD temporal cortex compared to controls was detected (Fig. 6a; *p* < 0.05). Furthermore, the concentrations of CCL2 and pGlu-CCL2 in temporal cortex of control and AD cases were quantified by ELISA. Interestingly, pGlu-CCL2 accounted for more than 90 % of total CCL2 in both conditions. In controls, 193.6 pg/ml CCL2 and 205.6 pg/ml pGlu-CCL2 were detected, indicating complete conversion of CCL2 into its pGlu-modified variant. In postmortem brains from AD subjects, pGlu-CCL2 levels were significantly higher than in control cases (Fig. 6a; *p* < 0.05) and represented 98.1 % of total CCL2 (451.2 pg/ml CCL2 and 442.7 pg/ml pGlu-CCL2).

### Correlation of isoQC and CCL2 with MMSE

To validate a possible role of isoQC in increased pGlu-CCL2 formation in human brain as well as in cognitive decline in AD, a series of correlation analyses plotting biochemical and clinical parameters from individual control and AD cases was performed.

Analyses revealed a strong correlation between increased levels of isoQC mRNA and low MMSE scores (*r* = −0.7220; *p* = 0.0080) and, similarly, between increased levels of CCL2 mRNA and low MMSE scores (*r* = −0.6124; *p* = 0.0343) (Fig. 6b, c).

However, correlation analysis revealed that there was no co-regulation of isoQC and CCL2 mRNA levels (Fig. 6d). Yet, CCL2 mRNA and protein levels displayed a strong positive correlation, validating the underlying measurements of mRNA and protein concentrations (Fig. 6e). Just as high isoQC mRNA and CCL2 mRNA concentrations, high pGlu-CCL2 protein levels strongly correlated with a decline in MMSE (*r* = −0.6336; *p* = 0.0270) (Fig. 6f).

Additionally, we investigated a possible interrelation between the amount of isoQC mRNA and the accumulation of Abeta and of pGlu-Abeta. While there was no correlation between isoQC transcript levels and total Abeta42 concentrations (Fig. 6g), a strong correlation between elevated isoQC mRNA levels and increased pGlu-Abeta became evident (*r* = 0.6663; *p* = 0.0180) (Fig. 6h). This provides further evidence for a role of isoQC in pGlu-Abeta formation, but not in Abeta generation in general.

## Discussion

In the present study, the expression pattern of isoQC in mouse brain is reported for the first time and we provide evidence for an implication of isoQC-driven CCL2 expression and stabilization in chronic inflammation typical for brains of AD patients and APP transgenic mouse models.

Using a highly specific affinity-purified antiserum, a surprisingly wide-spread distribution of isoQC protein expression in mouse brain was demonstrated. In particular, distinct and neuron-specific isoQC immunoreactivity was detected in many neocortical, hippocampal, subcortical and cerebellar structures of normal wild-type mice. Since brain-specific functions and substrates of isoQC are not yet known, this observation was unexpected and calls for an investigation of biological functions of isoQC in brain. The differential substrate specificity of QC and isoQC appears to arise from their distinct subcellular localization and not from enzymatic characteristics. An example is the thyrotropin-releasing hormone (TRH) tripeptide Gln-His-Pro which is generated from its precursor during maturation by prohormone convertases 1 and 3 and carboxypeptidase E/D in secretory granules [7, 14] and, therefore, is purely a substrate of QC, but not of Golgi-resident isoQC. On the other hand, the monocyte chemoattractant protein-1, CCL2, was demonstrated to be pGlu-modified and thus converted into a biologically active state in peripheral organs by isoQC, but not by QC [8].

The kinetic data shown here indicate that CCL2 is converted by isoQC, but with lower efficiency compared to smaller peptide substrates. Based on these experimental observations and on the reported role of CCL2 in AD [58, 60], we here addressed a possible interrelationship of isoQC and CCL2 in brain. Indeed, double immunofluorescent labeling revealed a co-localization of both proteins in mouse brain neurons and in cultured primary neurons. This co-localization of isoQC and CCL2 within intracellular compartments, i.e. at higher concentration of substrate and enzyme due to compartmentalization, might be important to facilitate the rather slow turnover of CCL2.

We also observed an up-regulation of isoQC and CCL2 mRNA levels and the induction of the enzyme isoQC and its putative substrate CCL2 in reactive astrocytes in proximity of Abeta plaques in Tg2576 mice. Interestingly, this astrocytic co-induction of both proteins followed a gradient from the plaque core towards the periphery, which suggests an Abeta-dependent mechanism. This co-induction of both proteins in the same cell type is indicative of a coordinated inflammatory response to recruit peripheral blood monocytes and other immune cells to loci of pathogenic protein aggregation. The resulting chronic gliosis may also contribute to aspects of AD pathology and to a decline in cognitive performance, a notion which is supported by a previous pharmacological treatment study. When young APP transgenic Tg2576 mice were treated with an inhibitor of QC/isoQC, pGlu-Abeta formation, total Abeta load and gliosis were reduced and memory deficits were ameliorated [53]. This is consistent with the view that prevention of pathogenic pGlu-Abeta seed formation has specific anti-inflammatory and mnemonic effects. However, when this pharmacological treatment was initiated in older mice—i.e. after initial pGlu-Abeta seed formation had already taken place—the total Abeta plaque load was not reduced, but accompanying microgliosis was ameliorated and context fear memory was improved in aged Tg2576 mice [53]. We hypothesize that the effects observed in this study can be attributed at least in part to inhibition of isoQC. Reduced enzymatic activity may then result in lowered astrocytic CCL2 pGlu modification, minimized CCL2 biological activity, alleviated microglial recruitment and diminished pro-inflammatory response, which collectively may lead to improved performance in the cognitive tasks tested. This scenario would be in line with recent studies demonstrating that an inhibitor of CCL2 synthesis protects neurons against Abeta toxicity [57] and that inhibition of microglial activation reduces cognitive deficits in a transgenic mouse model for AD [5].

The induction of AD-related proteins in reactive astrocytes following a number of challenges was consistently reported in the past for APP, BACE1 and γ-secretase components [17, 37, 64] and is expanded here to isoQC and its substrate CCL2. To corroborate this finding, we used a primary astrocyte model to study aspects of isoQC/CCL2 regulation under conditions of Abeta pathology. As a stimulation model, Abeta and pGlu-Abeta peptides were applied to astrocytic cultures. We observed a co-induction of both isoQC and CCL2 on the protein but not on the mRNA level. Under these conditions, there was a strong correlation between isoQC and CCL2 protein expression in individual culture wells, which was not observed for the transcript level. Thus, this model appears to be useful to study regulatory mechanisms of isoQC and CCL2 co-induction in astroglial cells. Additionally, astrocytes and microglia—but not neurons—were recently reported to be the source of N-terminally truncated Abeta peptides, another pathogenic QC/isoQC substrate [39].

Some of the observations made in APP transgenic Tg2576 mice were mirrored in brains of AD patients. In immunohistochemically processed sections of the temporal neocortex, a robust expression of isoQC in pyramidal neurons was conspicuous and much less pronounced in control cases. Additionally, isoQC was expressed by reactive astrocytes which also displayed CCL2 immunoreactivity in proximity to Abeta deposits. This indicates that similar mechanisms of isoQC and CCL2 regulation are active in the diseased human brain and in the transgenic mouse model analyzed. It is important to note that isoQC and CCL2 proteins were not detected in activated microglial cells of APP transgenic Tg2576 mice and AD subjects (data not shown).

Next, we sought to correlate the cognitive status of elderly humans with isoQC expression levels and pGlu-CCL2 protein levels in brain to assess the clinical significance of our findings. To address this issue, isoQC expression was quantified in temporal cortex (Brodmann Area 22), a part of the associative cortex involved in language processing [48]. Brain tissue with a very short postmortem delay and a well-documented clinical status was received from the Banner Health Brain Donation Program. Biochemical and molecular biological analyses included qRT-PCR to detect isoQC and CCL2 mRNAs as well as ELISA to quantify pGlu-Abeta and pGlu-CCL2 protein concentrations. Matching our immunohistochemical data, isoQC transcript levels were shown to be elevated in AD temporal cortex, as were total Abeta42 and pGlu-Abeta peptide concentrations and pGlu-CCL2 levels.

For individual cases, mRNA and protein measurements were correlated with the respective cognitive status as measured by MMSE. Here, a good correlation between isoQC mRNA levels and the concentration of pGlu-CCL2 was detected, supporting the notion of isoQC-catalyzed pGlu-CCL2 formation in human cortex contributing to pathological events. However, since only end-stage AD cases were analyzed in this study, it is not possible to draw conclusions on the sequence of events taking place. There were also strong inverse correlations between CCL2 mRNA and pGlu-CCL2 protein concentrations and MMSE. This is not surprising since neuroinflammatory events are characteristic of AD. On the other hand, CCL2 signaling is known to be enhanced in breast cancer, a condition present in the case AD3 of our cohort, which displays particularly high pGlu-CCL2 levels. Thus, the issue of possible co-morbidities should be addressed in further studies with larger cohorts. Taken together, this study points towards a specific role of isoQC and pGlu-CCL2 in cognitive decline in AD and underlines the therapeutic potential of targeting the generation of pGlu-CCL2 by inhibition of isoQC. Additionally, isoQC mRNA levels, but not QC mRNA concentrations inversely correlated with MMSE.

In recent studies, we have demonstrated a tight association between QC expression and pGlu-Abeta formation in hippocampus [15] and neocortex of AD patients [36]. There were strong correlations between QC mRNA levels and pGlu-Abeta concentrations and between pGlu-Abeta accumulation and a decline in MMSE [36]. Here, we expand these findings and provide additional evidence for a contribution of isoQC in pathogenic protein aggregation, neuroinflammation and cognitive decline in AD.

Based on the documented role of isoQC in peripheral inflammatory processes reported earlier and on the pro-inflammatory function of isoQC in brain demonstrated here for the first time, these findings point towards a critical role of inflammation in cognitive decline in AD. Together, our observations provide evidence for a dual involvement of QCs in AD pathogenesis by QC- and isoQC-catalyzed pGlu-Abeta formation and by isoQC-driven CCL2 stabilization maintaining chronic inflammatory events which collectively affect cognition.

## Electronic supplementary material

Below is the link to the electronic supplementary material.

Supplementary material 1 (DOC 8671 kb)
