# Supplementary material for: Isoglutaminyl cyclase contributes to CCL2-driven neuroinflammation in Alzheimer’s disease
Source: Acta Neuropathol. 2015 Feb 11;129(4):565–83. doi: 10.1007/s00401-015-1395-2 (PMC4366547; doi:10.1007/s00401-015-1395-2)
Supplement: Supplementary file 1 — Supplementary material 1 (DOC 8671 kb) [file 401_2015_1395_MOESM1_ESM.doc]

**Supplementary Information 1**

Isoglutaminyl cyclase contributes to CCL2-driven neuroinflammation

in Alzheimer’s disease

Maike Hartlage-Rübsamen, Alexander Waniek, Juliane Meißner, Markus Morawski, Stephan Schilling, Carsten Jäger, Martin Kleinschmidt, Holger Cynis, Astrid Kehlen, Thomas Arendt, Hans-Ulrich Demuth and Steffen Roßner

***Simultaneous induction of isoQC and CCL2 in mouse primary astrocytes upon LPS/IFN-γ stimulation***

To investigate the regulation of isoQC and CCL2 in astrocytes under general pro-inflammatory conditions not related to Abeta pathology, mouse primary astrocytes were treated with LPS, IFN-γ as well as a combination of both substances for different time periods and the mRNA and protein levels of isoQC and CCL2 were analyzed under control and experimental conditions. While isoQC protein was clearly detected in untreated astrocytes by immunocytochemistry, CCL2 protein was barely detectable (**Suppl. Fig. 1a**). Stimulation with LPS for 24 hours robustly increased the expression of both proteins, whereas only a minor effect of IFN-γ was observed (**Suppl.** **Fig. 1a**). However, the strongest up-regulation of isoQC and CCL2 was detected upon stimulation of astrocytes with the combination of both substances (**Suppl.** **Fig. 1a**).

A similar regulation of isoQC and CCL2 levels was measured by qRT-PCR at the mRNA level. The isoQC transcript levels increased by a factor of 1.8 after 12 hours of LPS/IFN-γ stimulation and then gradually declined over time (**Suppl.** **Fig. 1b**). CCL2 mRNA levels were up-regulated to an even larger extent (300-fold at 12 hours and 100-fold at 72 hours of LPS/IFN-γ stimulation). Interestingly, when plotting the individual isoQC versus CCL2 transcript levels of the same culture wells of astrocytes across all time points, there was no correlation under control conditions, but a highly significant correlation following stimulation with LPS/IFN-γ (**Suppl.** **Fig. 1c**). This is suggestive of a co-regulation of mRNAs for the enzyme isoQC and its substrate CCL2 upon LPS/IFN-γ stimulation.


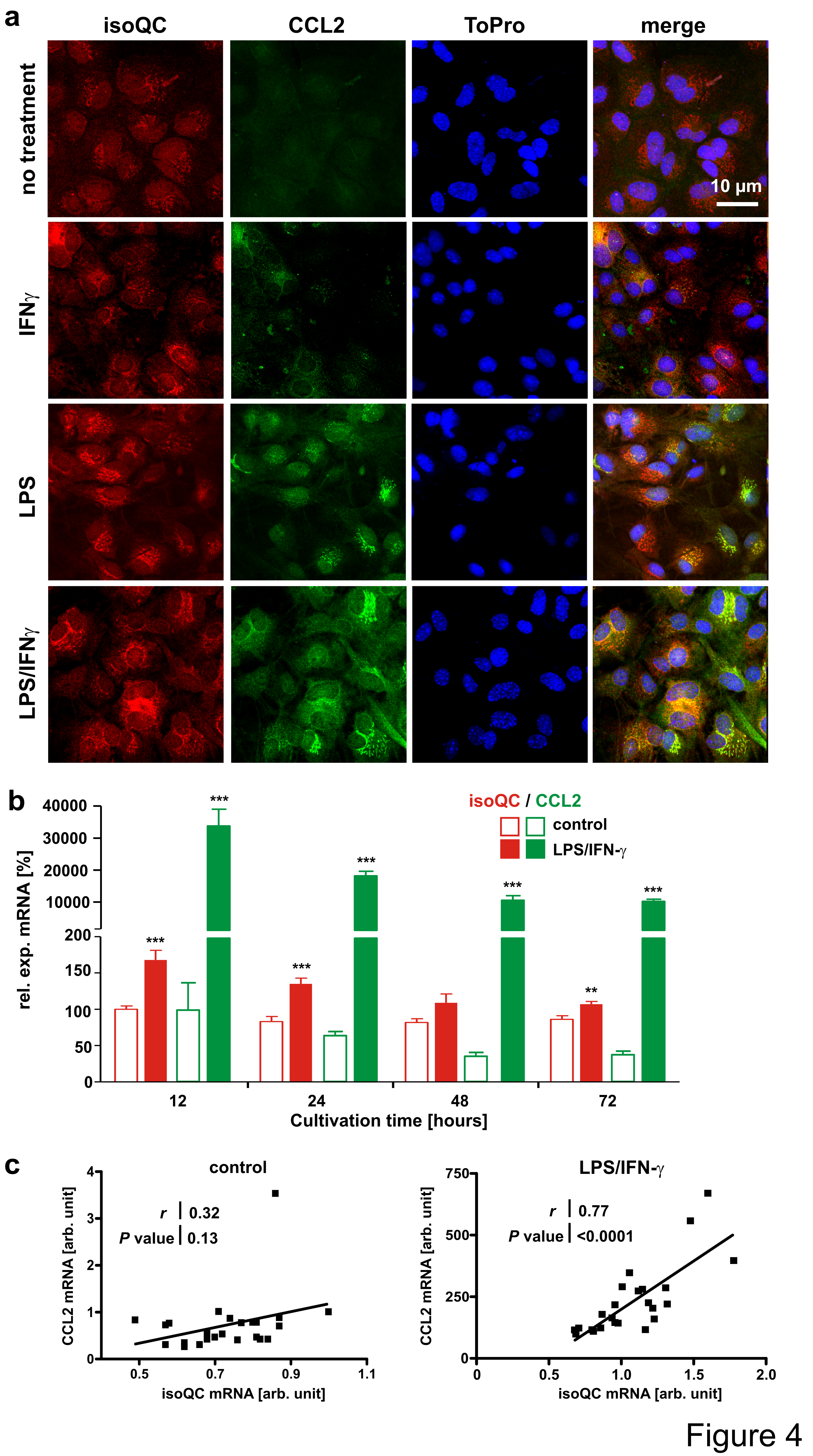


**Suppl. Figure 1:** *Co-regulation of isoQC and CCL2 in mouse primary astrocytes upon LPF/IFN-γ stimulation.*

**(a)** Immunocytochemical double labeling of isoQC (red) and CCL2 (green) with nuclear ToPro counterstaining under control conditions and after stimulation with LPS (1 µg/ml), IFN-γ (20 ng/ml) or a combination thereof as indicated. Note the robust increase in the immunocytochemical labeling intensity for both proteins and the subcellular co-localization of enzyme and substrate in Golgi-like structures. **(b)** Quantification of isoQC and CCL2 mRNA expression under control conditions and after stimulation with LPS/IFN-γ for different periods of time as indicated (N=6 per time point). Note the induction of isoQC enzyme mRNA levels by a factor of 1.8 and of the corresponding substrate mRNA levels by a factor of up to 300 at 12 hours after stimulation and the subsequent decline over time. **(c)** Correlation analyses of CCL2 mRNA levels plotted versus isoQC mRNA levels in individual astrocyte culture wells under control conditions and after LPS/IFN-γ stimulation including all time points analyzed. Note the absence of a correlation between enzyme and substrate mRNA expression levels under control conditions (left) and the highly significant correlation after LPS/IFN-γ stimulation (right).
